# A Metabolically-Stabilized Phosphonate Analog of Lysophosphatidic Acid Attenuates Collagen-Induced Arthritis

**DOI:** 10.1371/journal.pone.0070941

**Published:** 2013-07-29

**Authors:** Ioanna Nikitopoulou, Eleanna Kaffe, Ioanna Sevastou, Ivi Sirioti, Martina Samiotaki, Damian Madan, Glenn D. Prestwich, Vassilis Aidinis

**Affiliations:** 1 Institute of Immunology, Biomedical Sciences Research Center Alexander Fleming, Athens, Greece; 2 Echelon Biosciences Inc, Salt Lake City, Utah, United States of America; 3 Department of Medicinal Chemistry, University of Utah, Salt Lake City, Utah, United States of America; The University of Tennessee Health Science Center, United States of America

## Abstract

Rheumatoid arthritis (RA) is a destructive arthropathy with systemic manifestations, characterized by chronic synovial inflammation. Under the influence of the pro-inflammatory milieu synovial fibroblasts (SFs), the main effector cells in disease pathogenesis become activated and hyperplastic while releasing a number of signals that include pro-inflammatory factors and tissue remodeling enzymes. Activated RA SFs in mouse or human arthritic joints express significant quantities of autotaxin (ATX), a lysophospholipase D responsible for the majority of lysophosphatidic acid (LPA) production in the serum and inflamed sites. Conditional genetic ablation of ATX from SFs resulted in attenuation of disease symptoms in animal models, an effect attributed to diminished LPA signaling in the synovium, shown to activate SF effector functions. Here we show that administration of 1-bromo-3(S)-hydroxy-4-(palmitoyloxy)butyl-phosphonate (BrP-LPA), a metabolically stabilized analog of LPA and a dual function inhibitor of ATX and pan-antagonist of LPA receptors, attenuates collagen induced arthritis (CIA) development, thus validating the ATX/LPA axis as a novel therapeutic target in RA.

## Introduction

Rheumatoid arthritis (RA) is a destructive arthropathy with a high prevalence, imposing a substantial socioeconomic burden [1], [2]. Disease management for RA relies on symptomatic treatments that have relatively limited efficacy, can exhibit side effects and fail to improve long-term prognosis [3], [4]. The etiology and pathogenesis of RA remain poorly understood; however, it is widely accepted that the development of the disease requires a series of both autoimmune and inflammatory processes, mediated by a complex interplay between different cell types orchestrated by a dynamic cytokine network [1], [5], [6].

RA pathogenesis is characterized by the symmetric, chronic inflammation of the joints, eventually leading to the destruction of cartilage and bone [1]. Inflammation is initially localized in the synovial membrane, a thin layer of synovial cells lining diarthroidal joints that becomes markedly thickened due to synovial cell proliferation and infiltration by inflammatory cells [1], [5], [6]. Synovial fibroblasts (SFs) are among the dominant cell types of the arthritic synovium that, under the influence of the inflammatory milieu become activated and hyperplastic, while releasing a number of effector signals that include pro-inflammatory factors and tissue remodeling enzymes. While their role in disease initiation and regulation remains under investigation, activated SFs are widely recognized as major players in the manifestation and perpetuation of the disease [7], [8].

Autotaxin (ATX, ENPP2) is a secreted glycoprotein widely present in biological fluids, including synovial fluid [9], [10], [11]. ATX is a member of the ectonucleotide pyrophosphatase-phosphodiesterase family of ectoenzymes (E-NPP) that hydrolyze phosphodiesterase bonds of various nucleotides and derivatives [12]. More importantly, ATX is a plasma lysophospholipase D that catalyzes the conversion of lysophosphatidylcholine (LPC) to lysophosphatidic acid (LPA) [13]. In turn, LPA is a phospholipid mediator produced in various conditions both in cells and in biological fluids [11], [14], that evokes growth-factor-like responses in almost all cell types, including cell growth, survival, differentiation and motility [11]. The large variety of LPA effector functions is attributed to at least six, G-protein coupled, LPA receptors (LPARs) with overlapping specificities and widespread distribution [15], [16], [17].

ATX was originally isolated as an autocrine motility factor from the supernatant of melanoma cells [18]. Since then, increased ATX expression has been detected in a large variety of cancers and transformed cell lines [9], [11]. Beyond the established role of ATX in carcinogenesis [19], [20], high levels of ATX expression have been observed in non-malignant, inflamed tissues, suggesting a possible involvement of ATX in chronic inflammatory disorders, including RA [10]. Conditional genetic ablation of ATX from SFs results to attenuation of disease symptoms in animal models of RA [21], an effect attributed to diminished LPA signaling in the synovium, which, in turn, has been shown to activate effector function of SFs [21], [22].

Soon after the discovery of the potential roles of ATX and LPA signaling in carcinogenesis, many ATX inhibitors and LPAR antagonists were developed [23], [24], [25]. L-histidine and other metal chelating agents (ethylenediaminetetraacetic acid and 1,10-phenanthroline) were the first reported in vitro ATX inhibitors, based on their properties to scavenge metal ions essential for ATX activity [26]. Next, the discovery of product inhibition of ATX by LPA and S1P [27] led to the development of lipid-based ATX inhibitors including thiophosphates [28], analogs of cyclic phosphatidic acid (cPA) [29] and α-bromomethylene phosphonates [30], [31]. Finally, screening of compound libraries has resulted in the discovery of a number of small molecule, potent ATX inhibitors such as the boronic acid HA155 [32] and PF-8380 [33]. On the other hand, several LPAR antagonists have been reported, classified as lipid molecules mainly derived from LPA or cPA (Vinpocentins, diacylglycerol pyrophosphate) or non-lipid molecules (ki16425, AM095) [25].

1-bromo-3(*S*)-hydroxy-4-(palmitoyloxy)butyl-phosphonate (BrP-LPA), is an α-halo-substituted phosphonate, a metabolically stabilized analog of LPA that acts as a dual function pan-antagonist of LPA receptors and inhibitor of the lysophospholipase D activity of ATX [30]. BrP-LPA was shown to inhibit the invasiveness of transformed NIH3T3 cells expressing *ras* and ATX by 40% and to decrease their chemotaxis by 23% [31]; to reduce breast cancer cell (MDA-MB-231) migration *in vitro* and to cause tumor regression *in vivo* [34]; to inhibit cell migration and invasiveness of A549 cell *in vitro* and to inhibit tumor growth and angiogenesis in an engineered three-dimensional non-small cell lung cancer xenograft model [35]; to block the trans-differentiation of peri-tumor tissue fibroblasts and the associated proliferation, migration and invasion of hepatocellular carcinoma cells [36]; to reduce human colon cancer cells (HCT-116) proliferation, migration and invasion *in vitro* and to reduce liver tumor weight and volume *in vivo* [37]; and to reduce lymphocyte trafficking in draining lymph nodes [38]. Finally, JGW-8, a mixed isomer version of BrP-LPA, was shown to reduce synovial fluid-induced synovial fibroblast migration in a wound-closing assay [22].

The ligand properties of BrP-LPA were previously evaluated with Ca^++^ mobilization assays for accessing the activation and inhibition of LPA_1_, LPA_2_ and LPA_3_ in RH7777 cells, and LPA_4_ expressed in CHO cells [30]. These cell lines have been extensively used for the characterization of LPA GPCR ligands as RH7777 cells are intrinsically unresponsive to LPA, and CHO cells show minimal endogenous responses to LPA unless transfected with LPA_4_ [39], [40], [41]. BrP-LPA was able to recognize and inhibit all four receptors at different specificities (LPA_1_: 1.5 μM, LPA_2_: 1.4 μM, LPA_3_: 1.2 μM, LPA_4_: 0.27 μM) [30], establishing it as pan-antagonist of LPA receptors.

In this report, we first characterized the potency of BrP-LPA on the inhibition of ATX *in vitro* and *ex vivo* utilizing natural LPC substrates. The full pharmacokinetic profile of BrP-LPA *in vivo* was also determined showing that BrP-LPA rises to its highest level of 8 μΜ (C_max_) one hour (t_max_) post administration, accompanied by a parallel decrease in ATX activity levels (48% reduction) and LPA levels (82% reduction). Most importantly, intraperitoneal injections of BrP-LPA were able to prevent the arthritic symptoms in groups of mice undergoing the development of collagen induced arthritis (CIA), establishing the ATX/LPA axis as a novel therapeutic target in RA.

## Materials and Methods

### Animals

All mice were bred at the animal facilities of the Alexander Fleming Biomedical Sciences Research Center, under specific pathogen-free conditions. Mice were housed at 20–22°C, 55±5% humidity, and a 12-h light-dark cycle; water and food were given ad libitum. Mice were bred and maintained in their respective genetic backgrounds for more than ten generations. All experimentation was approved by the Institutional Animal Ethical Committee of Biomedical Sciences Research Centre Alexander Fleming (Re-Approval license No. 376), as well as by the Veterinary Service and Fishery Department of the local governmental prefecture. All efforts were made to minimize suffering during injections, and all surgery was performed in humanly sacrificed mice.

### Collagen induced arthritis (CIA)

CIA in DBA/1 mice was induced by immunization with collagen type II emulsified in complete Freund's adjuvant essentially as previously described [42], [43]. Mice were assessed for disease clinical signs two to three times per week. Clinical signs of arthritis were determined using the following scoring system [43]: 0: no evidence of erythema and swelling; 1: erythema and mild swelling confined to the tarsals or ankle joint; 2: erythema and mild swelling extending from the ankle to the tarsals; 3: erythema and moderate swelling extending from the ankle to metatarsal joints; 4: erythema and severe swelling encompassing the ankle, foot and digits, or ankylosis of the limb. As previously suggested [43], results are presented as the number of arthritic limbs per mouse, as well as mean score per arthritic limb.

### Histopathology and arthritic score

Paraffin-embedded joint tissue samples were sectioned and stained with hematoxylin and eosin as previously described [21], [44]. Arthritic histopathology in mice was assessed (in a blinded fashion from 3 independent examiners) using a semi-quantitative scoring system as previously described for CIA in a DBA/1 (H-2^q^) background. 0: no detectable pathology; 1: hyperplasia of the synovial membrane and presence of polymorphonuclear infiltrates – mild tendonitis may be present; 2: pannus and fibrous tissue formation and focal subchondrial bone erosion: 3: cartilage destruction and bone erosion in multiple foci; 4: extensive cartilage destruction and bone erosion- bone outline structure lost. Presented results refer to an average score of all four joints.

### Reagents

The various LPA and LPC species [myristroyl- (14:0), palmitoyl- (16:0), stearoyl- (18:0), oleoyl- (18:1), and arachidoyl- (20:4)] and their internal standards (17:1 LPC, LPA) were procured from Avanti Polar Lipids Inc. (Alabaster, AL, USA). Recombinant ATX(β) enzyme was obtained from R&D (Minneapolis, MN, USA), SinoBiological Inc (Beijing, China) and Echelon (Salt Lake City, UT, USA). HPLC solvents (methanol, chloroform, hexane, isopropanol), fatty acid–free BSA, chicken collagen type II, choline chloride, 4-aminoantipyrene (4-AAP) and horseradish peroxidase were obtained from Sigma (Sigma-Aldrich, St. Louis, MO, USA). Choline oxidase was from MP Biomedical (Santa Ana, CA, USA), TOOS reagent was from TCI Europe (Antwerp, Be), Amplex Red was from Invitrogen (Carlsbad, CA, USA) and FS-3 was from Echelon.

### HPLC-MS/MS

BrP-LPA, LPA species (C14:0, C16:0, C18:0, C18:1 and C20:4) and LPC species (C14:0, C16:0, C18:0, C18:1 and C20:0) were measured in plasma with HPLC-ESI/MS/MS using RSLCnano system (Ultimate 3000) coupled in-line with an LTQ Orbitrap XL mass spectrometer (Thermo Scientific, Waltham, MA, USA). BrP-LPA and lipid (LPA, LPC) extraction from plasma was performed on ice according to the Folch method with minor modifications [45]. Plasma samples (50 ul) were mixed with 950 ul PBS prior to extraction and spiked with the internal standard mix (17:1 LPA/LPC). Samples were extracted under neutral conditions with 4 ml of ice-cold CHCl_3_/CH_3_OH (2/1, v/v) followed by 2 ml of PBS-saturated ice-cold CHCl_3_/CH_3_OH (2/1, v/v). Each extraction step was followed by a 60 s vortex and a 1 min centrifugation step in a bench-top clinical centrifuge in a cold room at 4°C at 3,000 rpm. The lower chloroform organic phases from both neutral extraction steps were pooled and kept for LPC measurement. The remained aqueous phase was chilled on ice for 10 min and extracted twice with ice-cold acidified with acetic acid CHCl_3_/CH_3_OH (2/1, v/v) as above. The lower organic phases were pooled, neutralized to pH 6–7 (checked with pH paper as above) on ice and kept for LPA, BrP-LPA measurements. The kept neutral lower organic phase of neutral extraction and the neutralized acidified lower organic phase were evaporated to dryness. The resulting residues were resuspended in 0.15 ml of isopropanol and after brief vortex mixing the solution was transferred to the auto sampler vial. Recovery of LPA species was between 60% and 95%. Recovery of BrP-LPA was 70% and the recovery of LPC species was between 80% and 100%. The Relative Standard Deviation (RSD) was less than 7% from the plasma fortified with three different concentrations (0.1, 0.5 and 1 μΜ) of LPAs, (0.1, 1 and 10 μΜ) of BrP-LPA and (0.5, 5 and 50 μΜ) of LPCs. Analysis was performed using a Phenomenex (Phenomenex, Torrance, CA, USA) Luna Silica column (2–250 mm, 5 μm particle size) using a binary gradient program consisting of isopropanol:hexane:100 mM NH_4_CO_2_H(aq) 58:40:2 (mobile phase A) and isopropanol:hexane:100 mM NH_4_CO_2_H(aq) 50:40:10 (mobile phase B). The mobile phase was delivered at a flow rate of 0.3 ml/min and the total run time was 50 min/sample. The analysis was performed with electrospray ionization in negative SIM (Single Ion Monitoring) mode for LPA species and BrP-LPA and in positive mode for LPC species (data dependent MS/MS). The resolution was 100 K providing high accuracy for lysophospholipid measurements. Data were collected and processed with the Xcalibur software (Thermo Scientific). The precursor ion mass was used for the quantification while lipid identity was confirmed by precursor ion fragmentation using collision induced dissociation (CID). The peak area ratios (lipid/internal standard) versus the molar ratios (lipid/internal standard) were plotted and fitted to a linear regression. The linear dynamic range of LPA species and BrP-LPA was between 0.01 μM and 10 μΜ.

### TOOS Activity Assay

ATX/LysoPLD activity was assessed based on the amount of choline released from LPC with the use of TOOS (N-ethyl-N-(2-hydoroxy-3-sulfopropyl)-3-methylaniline) reagent. In this assay ATX-mediated LPC-liberated choline is oxidised by choline oxidase to betaine and hydrogen peroxide (H_2_O_2_). The latter in the presence of horseradish peroxidase reacts with TOOS and 4-aminoantipyrene (4-AAP) to form a pink quinoneimine dye measured at 555 nm [13].

Diluted plasma samples (100 fold dilution; final EDTA concentration 0.5 mM) or recombinant ATX protein (2 nM) were incubated with LPC in the presence of 100 mmol/l Tris–HCl/pH 9.0, 500 mmol/l NaCl, 5 mmol/l MgCl_2_, 5 mmol/l CaCl_2_, 60 μM CoCl2 and 0.05% Triton X-100 for 3 h at 37°C at a final volume of 100 μl. It should be noted that EDTA was used not only as an anticoagulant but also as an inhibitor of ATX activity *ex vivo* [46], in order to suppress artificial LPA production during blood withdrawal and plasma preparation [47]; this is because the same samples were used in phospholipid measurements. The inhibitory effect of EDTA is reversed in the enzymatic assay by the addition of 5 mM of each MgCl_2_ and CaCl_2_ as previously reported [26], as well as by the addition of cobalt ions that were shown to stimulate ATX activity [48].

The liberated choline was detected by adding 100 μl of a color mix containing 4-AAP and TOOS (0.3 mM), horseradish peroxidase (5.3 UnitmL^−1^) and choline oxidase (2 UnitmL^−1^). Absorbance was measured at 555 nm and data were analyzed using the statistical software package Prism 4.0 (Graphpad Software Inc, La Jolla, CA, USA). For each sample and control the absorbance was plotted against time and the slope (da/min) for the linear (steady-state) portion of each reaction profile was calculated. Absorbance units were converted to nmol of choline/min/ml by comparison with a choline standard curve or by use of the extinction coefficient for the quinoneimine dye according to the following equation:
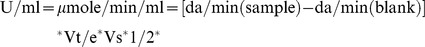
Where Vt* is the total volume of reaction in ml (0.2 ml); Vs* is the volume of sample in ml; e is the millimolar extinction coefficient of quinoneimine dye under the assay conditions (e =  e = 32.8 μmol/cm^2^); and 1/2: Factor based on the fact that 1 mol of hydrogen peroxide produces 1/2 mol of quinoneimine dye.

### FS-3 ATX activity assay

Inhibition of ATX activity by BrP-LPA was determined using the cleavage of the ATX-specific fluorogenic substrate FS-3 (Echelon Biosciences, UT, USA). Assays were conducted in a final volume of 100 μl in the presence of 140 mM NaCl, 5 mM KCl, 1 mM MgCl_2_ and 50 mM Tris, pH 8.0 with the use of 1 µM FS-3, 2 nM ATX from Echelon and increasing concentrations of BrP-LPA. Reactions were brought up to 100 µl with ddH_2_O and were then incubated at 37°C in a Tecan Infinite 200 microplate reader (Tecan Trading AG, Switzerland) set to make fluorescence measurements every 1 min for a total period of 30 min. Autotaxin activity was quantified by measuring the rate of increase in fluorescence at 528 nm with excitation at 485 nm. At the end of the measurement, each data point was plotted on a graph of fluorescence versus time. A linear regression was fitted in the linear portion of the graph (3–20 min).

### Amplex Red Activity Assay

BrP-LPA inhibition was also tested with the more sensitive reagent Amplex Red based on the Amplex™ Red PLD assay kit (Molecular Probes, Interchim, Montluçon, France) originally designed for the measurement of the PLD activity [46], [49]. All reactions were performed with purified ATX (2 nM) from Echelon at 37°C. The ability of ATX to cleave LPC was detected using a coupled assay that monitors the release of choline using Amplex Red (10 μΜ), choline oxidase (0.2 Units/ml) and horseradish peroxidise (2 Units/ml) The rate of fluorescence increase was measured in a fluorescence plate reader (Tecan Infinite 200 microplate reader from Tecan Trading AG, Switzerland) for up to 80 minutes; the fluorescence increase was linear during that time-frame for all cases. Rates were normalized to control reactions that contained all reaction components except for the test compound.

### ATX Kinetic Assays

The biochemistry of lyso-PLD was assessed by following the kinetics data using a wide panel of different ATX substrates [FS-3, different LPC species (14:0, 16:0, 18:0, 18:1)], three different recombinant ATX proteins (R&D, Sino-Biological and Echelon) and three different ATX activity assays (FS-3, TOOS, Amplex Red). The ATX activity was measured at different substrate concentrations (typically from 1-1000 μM) in order to examine whether ATX follows Michaelis-Menten kinetics. The Michaelis constant (K_m_) was obtained by fitting the substrate dependence of V (ATX activity) to a rectangular hyperbola using the non-linear regression analysis of the statistical software package Prism 4.0 (GraphPad Software, Inc.). K_m_ and V_max_ were evaluated from the rectangular hyperbola curve with the use of GraphPad software.

### *In vitro* Inhibition Assay

For *in vitro* inhibition measurements, BrP-LPA was dissolved in water as a stock solution (4 mM) and appropriate dilutions were made in water. The inhibition of ATX by BrP-LPA was measured employing 2 nM recombinant ATX, 50 μΜ LPC (14:0, 16:0, 18:0) and 0.01 to 100 μmol/L of BrP-LPA (final concentrations). The *in vitro* inhibition of ATX by BrP-LPA was evaluated with three different assays (FS-3, Amplex Red, and TOOS). To ensure that BrP-LPA did not interfere with the colouring agent or hydrogen peroxide that is generated during the colour forming reaction in Amplex Red and TOOS activity assay, samples with BrP-LPA were incubated with only choline and the colouring reagents were added subsequently. BrP-LPA was also tested for its ability to inhibit mouse plasma ATX activity. Diluted mouse plasma was used as the source of ATX and the analysis of inhibition was made with TOOS as the *in vitro* inhibition assay.

### Inhibition Analysis

The inhibition model (competitive, noncompetitive, or mixed type inhibition) was evaluated by graphical analysis with Lineweaver–Burk double reciprocal plots (1/[velocity] versus 1/[substrate]) and Cornish-Bowden plot ([substrate]/[velocity] versus [Inhibitor]). These plots were generated by the measurement of ATX activity in the presence of different BrP-LPA concentrations (0–10 μΜ) and increased amounts of 16:0 LPC (100, 200, 400 μΜ). The inhibition constants (K_i_) and IC_50_ values were determined using the mean values obtained from two independent experiments. The relative remaining ATX activity or LPA levels in the presence of the BrP-LPA was expressed as a percentage of the corresponding control value (ATX activity, LPA levels without inhibitor). The IC_50_ value was determined graphically by plotting the log concentration of BrP-LPA versus relative remaining ATX activity or LPA levels of each test sample using the sigmoid dose–response fitting method (Prism^®^ software). The IC_50_ (concentration of BrP-LPA sufficient to inhibit ATX activity or diminish LPA levels by 50%) was calculated by the equation f =  y0+a/(1+exp(-(x-x0)/b)) given by SigmaPlot 11.0 (Systat Software, Inc., San Jose, CA, USA).

K_i_ was calculated using the equation of Cheng and Prusoff: Ki = IC_50_/1+[S]/K_m_ [50], where K_i_ is the binding affinity of the inhibitor, IC_50_ is the functional strength of the inhibitor, [S] is substrate concentration and K_m_ is the concentration of substrate at which enzyme activity is half maximal. Whereas the IC_50_ value for a compound may vary between experiments depending on experimental conditions, K_i_ is an absolute value. Alternatively the Ki values were calculated graphically as follows: The velocity of ATX (v) was determined at two or more substrate concentrations and over a range of inhibitor concentrations (I). In a plot of 1/v against I, data for each substrate concentration fall on straight lines that intersect at I = −K_i_ and 1/v = 1/V_max_ (competitive inhibition) and at I = −K_i_ and 1/v = [1−(K_i_/K_i_')]/V_max_ (mixed type inhibition).

### Inhibition of ATX Activity and LPA Production in Whole Blood *ex vivo*

Venous blood from wild type mouse was collected in EDTA tubes. Blood was incubated with BrP-LPA at different final concentrations (0–10 μM) in 96-well collection plates for 2 h at 37°C. The reaction was stopped by centrifugation at 1000 *g* for 10 min to pellet the cells, which has been reported to remove most platelets [51]. Plasma supernatant was kept for the measurement of ATX activity and LPA/LPC species with TOOS activity assay and HPLC/MS/MS respectively. IC_50_ values using ATX activity and LPA levels were determined graphically as described above.

### Mouse Pharmacokinetics and *in vivo* Inhibition of ATX Activity and LPA Production

BrP-LPA was administered intraperitoneally (IP) at 10 mg/kg in water to 6–8 week old female mice. Control mice (n = 3) received only the vehicle (water). Blood samples were collected from each animal (n = 3 animals per time point) via a cardiac puncture at time points up to 16 hours post administration. Plasma was prepared by centrifugation of whole blood at 1000 g for 15 min at 4°C, and was stored frozen (−80°C) in siliconized tubes. Plasma samples were then analysed for plasma ATX activity and BrP-LPA/LPA/LPC levels with the TOOS activity assay and HPLC-MS/MS respectively. Maximum plasma concentrations (C_max_), their time of occurrence (T_max_) and half-life (t_1/2_) were both obtained directly from the acquired data.

### Statistical Analysis

Statistical significance of all values, always in pair-wise comparisons with control values, was assessed with a paired Student's *t* test, following confirmation of normal distribution with SigmaPlot 11.0. p values <0.05(*), p<0.01 (**) and p<0.001 (***) were considered significant. For CIA clinical scores the Mann-Whitney rank sum test was used to determine the level of significance between means of groups.

## Results and Discussion

### BrP-LPA inhibits the ATX-mediated hydrolysis of natural LPC substrates *in vitro*

In addition to its antagonistic effect on LPA receptors, BrP-LPA was shown to inhibit the lysophospholipase D activity of ATX (94% inhibition at 10 μM) [30], using the substrate FS-3, a fluorogenic doubly labeled LPC analog, wherein the fluorophore is quenched through intramolecular energy transfer [52]. FS-3 has been used extensively for the discovery of novel ATX inhibitors [29], [33], [53], [54], [55]; however, FS-3 is an artificial ATX substrate that behaves very differently from LPC in terms of binding to ATX and saturation kinetics [56], [57]. Therefore, we sought to determine the ability of BrP-LPA to inhibit hydrolysis of natural LPC substrates by recombinant ATX (from Sinobiological Inc), by measuring the released choline in a colorimetric enzyme-coupled reaction utilising the TOOS assay.

**Figure 1:**
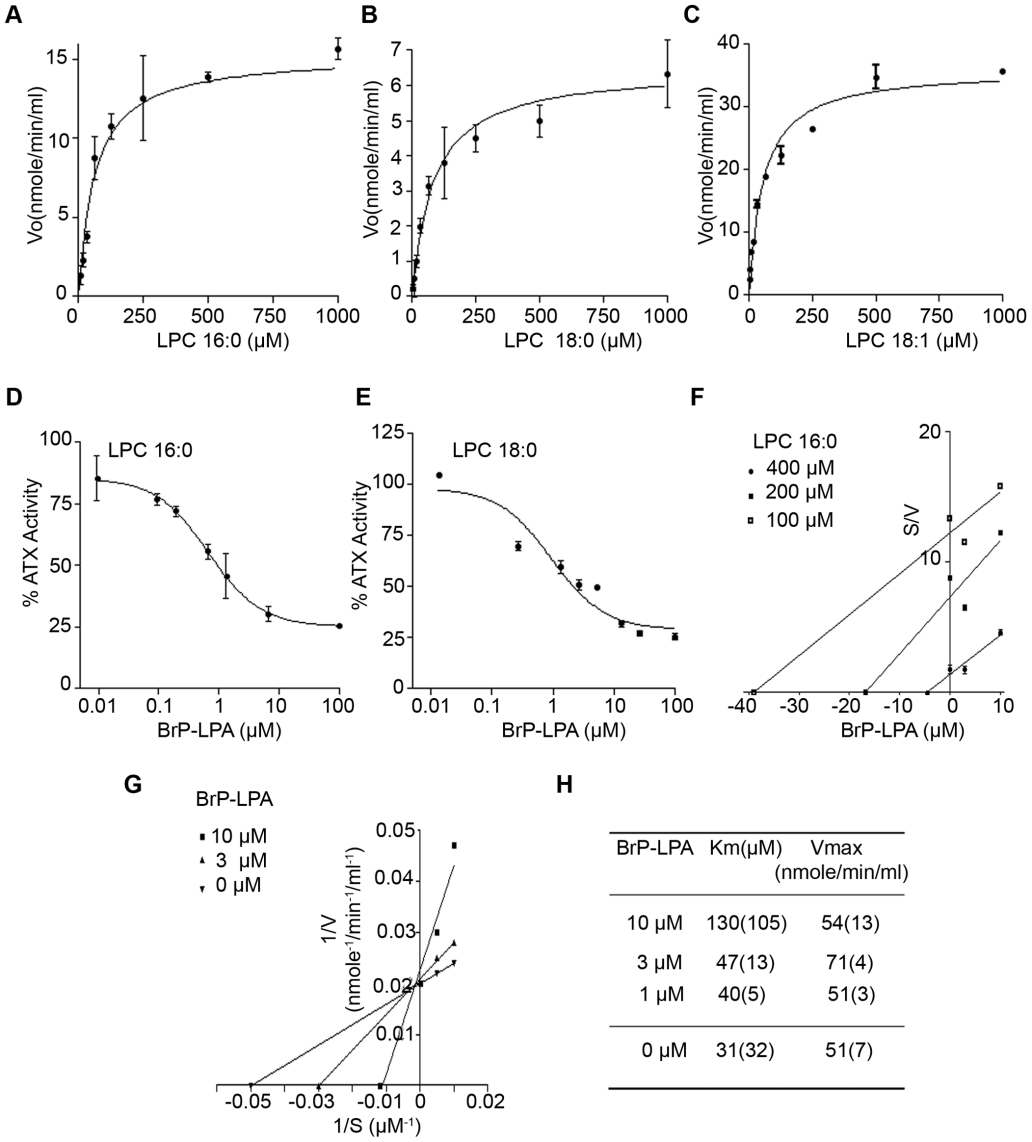
BrP-LPA inhibits the ATX-mediated hydrolysis of natural LPC substrates *in vitro*. **A–C.** Steady-state hydrolysis of LPC (16:0, 18:0, 18:1 respectively) by recombinant ATX as measured with the TOOS activity assay. **D–E.** ATX activity inhibition dose-response curves in the presence of various BrP-LPA concentrations as measured with the TOOS assay using as substrates 50 μΜ of 16:0 (D) and 18:0 LPC (E). **F–G**. Cornish-Bowden (F) and Lineweaver-Burk (G) plots show that BrP-LPA is a competitive ATX inhibitor. **H.** The kinetic parameters (k_m_, V_max_) obtained by the incubation of ATX at various concentrations of 16:0 LPC (100–400 μΜ) in the absence or presence of BrPLPA (0–10 μΜ). All presented values are the means (±std) of two independent experiments.

The time course of ATX-dependent LPC (16:0) hydrolysis was found linear within the first 20 minutes (data not shown), so all subsequent analyses were performed at this time point. The hydrolysis rate (V_o_) of LPC 16:0, 18:0 and 18:1 by ATX was found to depend hyperbolically on the LPC concentration and to follow Michaelis-Menten kinetics (Figure 1A, B, C). The K_m_ for each substrate was then determined, as detailed in the Material and Methods section. ATX was found to have the highest affinity for 18:1 LPC (K_m_ = 55 μM), followed by 16:0 LPC (K_m_ = 60 μM) and 18:0 LPC (K_m_ = 82 μM) (Table 1). Similar results in substrate affinity order were obtained with a different recombinant ATX protein (from R&D) (Fig. S1A, B and Table 1). Moreover, similar results were also obtained with yet another source of recombinant ATX (Echelon) and a different colorimetric assay (Amplex Red; Fig. S1C, D and Table 1). In comparison, the K_m_ of ATX for FS-3 was found to be much lower than natural LPC substrates (Fig. S1E and Table 1), confirming previous studies suggesting that the modification of the choline moiety in FS-3 results in a much higher affinity and tighter binding than natural LPC substrates [55]. The K_m_ of ATX for the different species of LPC was found to be in the range of 50–150 μΜ, in agreement with previously reported values [23], [32], [58], [59], [60], spanning the range of physiological LPC levels in plasma [61], [62], and well above the critical micelle concentration (CMC) of all LPC species used [63]. Moreover and as previously reported, our results indicate that ATX prefers unsaturated LPC species and that the length of the LPC acyl chain is crucial for the affinity of LPC with ATX [58], [64].

**Table 1:** Kinetic parameters of *in vitro* LPC hydrolysis by recombinant ATX and inhibition constants of BrP-LPA.

LPC species	16:0	18:0	18:1	14:0	18:0	FS-3
Assay	TOOS	TOOS	TOOS	Amplex Red	Amplex Red	FS-3
Km (μΜ)	60±11 *90±21* *	82±11 *114±34* *	55±10	80±9	116±13	2.3±0.4
IC_50_ (μΜ)	0.7±0.2	0.9±0.1	-	1.6±0.1	1.1±0.1	0.4±0.03
[S] (μΜ)	50	50	-	50	50	1
% Inhibition at 10 μΜ	80%	70%	-	75%	75%	95%
Ki (μΜ)	0.7±0.1	0.6±0.11	-	1.0±0.4	0.8±0.2	0.3±0.1

The presented values are the means (±std) of two independent experiments.

* *Km values for recombinant ATX from R&D*.

The ability of BrP-LPA to inhibit the lysoPLD activity of ATX was then evaluated using different LPC species as substrate, at concentrations [S] below the corresponding K_m_ values. A clear dose response effect on ATX inhibition was observed for BrP-LPA for all LPC species (16:0, 18:0) tested (Fig. 1D, E). Similar results were obtained with the Amplex Red and FS-3 assays (Fig. S1F, G, H). IC_50_ and K_i_ values were then calculated as described in the Materials and Methods section and are reported in Table 1. Similar K_i_ values were obtained graphically from Dixon plots (data not shown). Moreover, the mode of inhibition was determined from the inhibitory effect of BrP-LPA on K_m_ and V_max_ values with 16:0 LPC as a substrate. BrP-LPA was found to inhibit ATX lysoPLD activity in a competitive manner from Cornish-Bowden (Fig. 1F) and Lineweaver-Burk (Fig. 1G) plots, as V_max_ remained unchanged and K_m_ increased at different BrP-LPA concentrations (Fig. 1H).

### BrP-LPA inhibits ATX activity *ex vivo*

**Figure 2:**
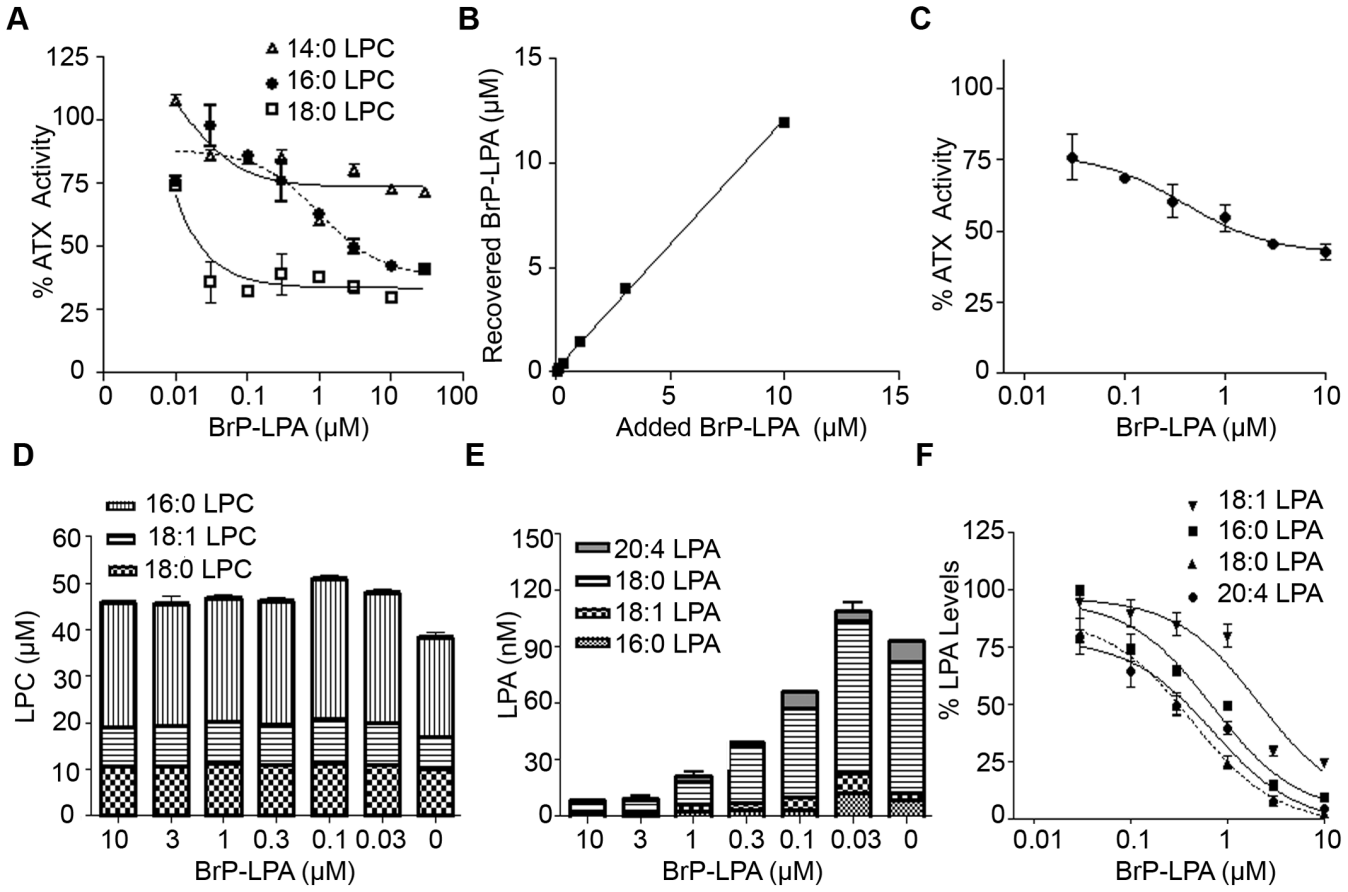
BrP-LPA inhibits ATX activity *ex vivo*. **A.** Effect of BrP-LPA on plasma ATX/lysoPLD hydrolysis of exogenous added 50 μΜ LPC (14:0, 16:0, 18:0). **B.** Correlation of recovered BrP-LPA in whole blood with the added BrP-LPA. **C.** Inhibition of endogenous ATX/lysoPLD activity in whole blood *ex vivo* after the addition of increasing BrP-LPA concentrations (0–10 μΜ). ATX activity was measured in the presence of 1 mM LPC with the TOOS assay. **D.** LPC levels and **E.** LPA levels measured in whole blood ex vivo in the absence/presence of different BrP-LPA concentrations (0.03–10 μΜ). **F.** Per cent residual levels of the indicated LPA species in the presence of increasing amounts of BrP-LPA. Solid lines, best fits of averaged data points. * indicates significant (p<0.05), *** indicates significant (p<0.001) decrease relative to control group.

ATX is identical to plasma lysoPLD [13] and responsible for the majority of LPA production in plasma [65]. Therefore, BrP-LPA was then tested for its ability to inhibit endogenous ATX/lysoPLD activity in plasma *ex vivo*. Increasing amounts of BrP-LPA were added to mouse plasma *ex vivo* and ATX activity was measured with the TOOS assay utilizing different exogenous natural LPC substrates at final concentrations of 50 μM. In agreement with the *in vitro* ATX activity inhibition studies, BrP-LPA also inhibited plasma ATX/lysoPLD activity (Fig. 2A). Moreover, BrP-LPA mediated inhibition of exogenous-LPC hydrolysis by ATX appeared to be more potent when low affinity LPC species were used (18:0>16:0>14:0; Table 2). Next, the ability of BrP-LPA to inhibit the hydrolysis of endogenous LPC species in whole blood *ex vivo* was examined. A direct correspondence of added and recovered BrP-LPA was observed (Fig. 2B), thus corroborating the experimental procedures. The effect of BrP-LPA on ATX activity was found to be dose-dependent with an IC_50_ of 600 nM (Fig. 2C).

**Table 2:** Inhibition constants as measured by dose-response curves obtained by residual percent activity of plasma ATX/LysoPLD (TOOS activity assay) after the addition of increasing amounts of BrP-LPA (0.01–100 μM) in the presence of different exogenous LPC species.

	14:0 LPC	16:0 LPC	18:0 LPC
IC_50_ (μΜ)	>10	2.9±0.3	<0.1
[S] (μΜ)	50	50	50
% Inhibition at 10 μΜ	28%	58%	70%

The presented values are the means (±std) of two independent experiments.

In the same experiments no significant changes were observed in the endogenous LPC levels upon the addition of BrP-LPA (Fig. 2D). This is consistent with the unchanged radiolabeled LPC levels upon the addition of the potent, boronic-acid based, ATX inhibitor ex vivo [32], and confirms that ATX inhibition does not appreciably affect the large pool of plasma LPC. In agreement, heterozygous, complete knock out (hKO) mice expressing 50% of ATX did not present with increased LPC levels (data not shown and [66]). On the other hand and most importantly, reducing ATX activity *ex vivo* with BrP-LPA resulted in lower LPA levels, with the highest potency observed at 10 μM, reaching 86% reduction of total LPA levels and 97% reduction of 18:0 LPA levels (Fig. 2E, F). The effect of BrP-LPA on the levels of the most abundant LPA species (18:0>20:4>16:0>18:1) [67], [68] was found to be dose-dependent (Fig. 2F), while BrP-LPA inhibited more potently the production of 18:0 LPA (IC_50_ = 300 nM) followed by 20:4 LPA (IC_50_ = 559 nM), 16:0 LPA (IC_50_ = 1340 nM) and 18:1 LPA (IC_50_ = 1520 nM).

### BrP-LPA inhibits ATX activity *in vivo*

**Figure 3:**
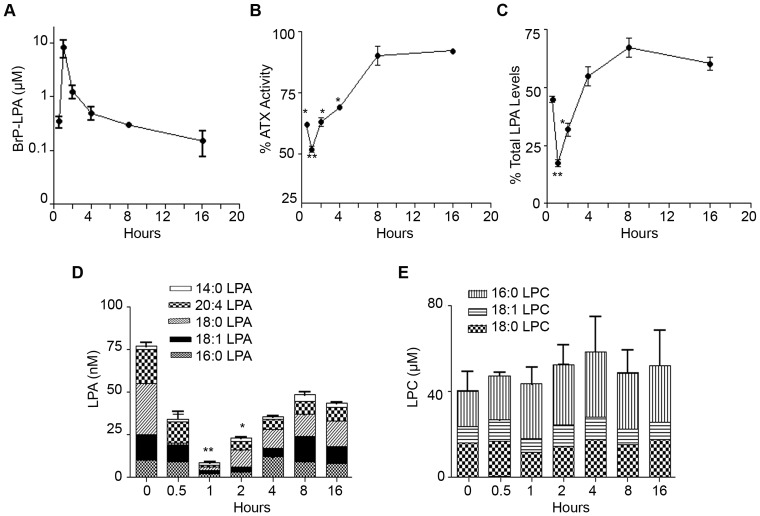
BrP-LPA inhibits ATX activity *in vivo*. **A.** Plasma BrP-LPA pharmacokinetic profile following i.p administration of 10 mg/Kg BrP-LPA in female mice. **B.** Plasma per cent (%) residual ATX activity measured in the presence of 1 mM LPC with the TOOS assay and **C**. Plasma % residual total LPA levels at different time points after i.p. administration of BrP-LPA. **D.** Plasma concentration of different LPA and **E.** LPC species at different time points following BrP-LPA i.p administration. The time point 0 refers to the vehicle control. The presented values are the means (±std) of two independent experiments. *(p<0.05) and **(p<0.01) indicate a significant decrease relative to control group.

To investigate how BrP-LPA affects plasma ATX activity and LPA levels *in vivo*, we next administered BrP-LPA (10 mg/kg) or vehicle alone as a single intra-peritoneal bolus injection to groups of mice. The levels of BrP-LPA, ATX activity, LPA and LPC were then determined in plasma 0.5, 1, 2, 4, 8 and 16 h post injection. The pharmacokinetic (PK) analysis of BrP-LPA shows that it rose to its highest level of 8 μΜ (C_max_) at 1 h (t_max_) post-administration and declined rapidly (t_1/2_ = 50 min, C_min_ = 0.15 μM at 16 h) (Fig. 3A). The pharmacodynamic (PD) effect of BrP-LPA was measured as decreasing ATX activity and LPA levels in plasma: BrP-LPA reduced ATX activity (48%; Fig. 3B) as well as total LPA (82%; Fig. 3C) levels 1 hour post administration, where it exhibited its maximum inhibitory effect. Sixteen hours post administration ATX activity returned near baseline (92% of initial activity; Fig. 3B) while LPA levels returned at 60–70% of normal initial levels (Fig. 3C, D). BrP-LPA inhibited the production of all LPA species tested except from 14:0 LPA (Fig. 3D), possibly due to the high affinity of 14:0 LPC for ATX. As expected from the low *ex vivo* IC_50_ values, the inhibitory effect of BrP-LPA on 18:0/20:4 LPA production was longer lasting than for other LPA species. LPC levels remain unchanged as expected from the *ex vivo* experiments (Fig. 3E).

### BrP-LPA attenuates collagen induced arthritis

ATX levels have been found to be elevated in the synovial fluids of RA patients, as well as in joint tissues and sera of both RA patients and arthritic models [21]. Moreover, ATX has been shown to be predominantly expressed and secreted from arthritic SFs, both in human patients and mouse models [21]. ATX expression from SFs was shown to be induced by TNF [21], the major pro-inflammatory factor driving RA development, which has also been shown to induce NF-κB-mediated ATX expression in hepatocytes [69], suggesting that ATX induction in arthritic SFs is a downstream event of exacerbated TNF signaling in the synovium. Conditional genetic ablation of ATX from SFs results in attenuation of disease symptoms in animal models of RA [21] establishing ATX as a novel player in the pathogenesis of RA.

ATX induction in the synovium and other inflammatory sites is likely to stimulate local LPA production and its autocrine and paracrine effects. This hypothesis was tested in a rat air pouch model, where potent ATX inhibition provided >95% reduction in air pouch LPA, indicating ATX as the major source of LPA at inflammatory sites [33]. Mouse SFs have been shown to express all five major LPARs (1–5) [21], while human SFs were reported to express LPARs 1–3 [70], [71]. LPA has been shown to stimulate SFs actin cytoskeleton rearrangement [21], a pathological determinant of RA [44], in accordance with the reported functions of LPA in fibroblasts [72]. Moreover, LPA has been shown to stimulate SF proliferation, adhesion, migration, cytokine and MMP production [21], all characteristics of the aggressive behavior of arthritic SFs [44], [73], [74]. The effects of LPA in SF activation have been shown to be dependent on G-protein and MAPK signaling and to synergize with TNF in SF activation [21]. LPA (together with PDGF) has been reported to mediate migration in a tenascin-C substratum, shown to be essential for maintaining inflammation in arthritic joints [75]. LPA is also known for altering cell-to-cell contacts [11], which have been found to be crucial for RA pathogenesis [76]. Moreover and beyond the effects of LPA to stromal cells, LPA has been shown to stimulate polarization, motility and transendothelial migration of naive T-cells [77], suggesting that LPA can also actively modulate lymphocyte homing. Therefore, in the context of disease pathogenesis, LPA stimulates a number of inflammatory pathways and SF effector functions necessary for RA development.

**Figure 4:**
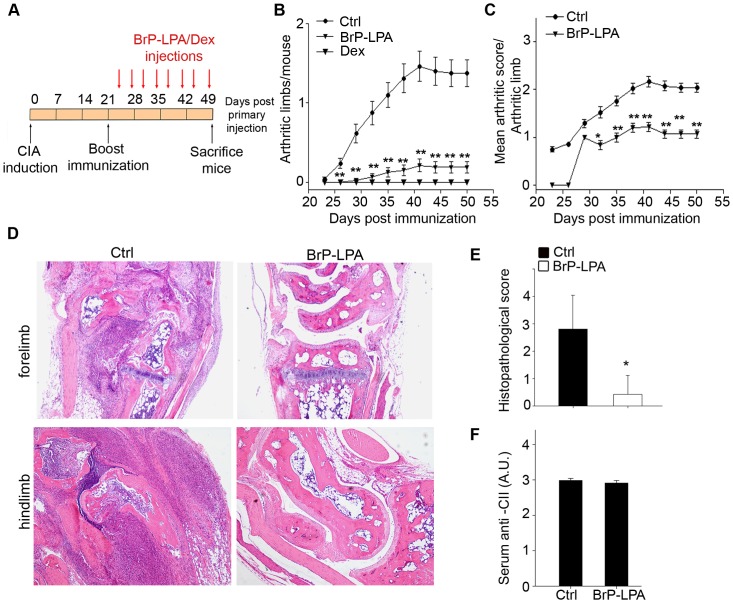
BrP-LPA attenuates collagen induced arthritis. **A.** Schematic representation of immunization and drug administration schemes. DBA/1 mice subjected to CIA were treated intraperitoneally with vehicle, BrP-LPA (10 mg/kg), or dexamethasone (Dex, 3 mg/kg) twice a week. **B–C.** Time dependent clinical scores (± SEM) after primary immunization with CII. Data are pooled from two different experiments (n = 10). **D.** Representative sections of the forelimb and hindlimb joints of vehicle-treated and BrP-LPA-treated mice, stained with H&E. **E.** Quantification of disease severity in vehicle-treated and BrP-LPA-treated mice. Joint sections were assessed histologically in a blinded manner by three independent examiners. Data are shown as mean joint values per mouse ± SD. **F.** Humoral response to collagen in vehicle-treated or BrP-LPA-treated mice. Levels of CII-specific Abs were determined by ELISA in mouse sera. Results show the mean ± SEM values. *(p<0.05) and **(p<0.01) indicate a significant decrease relative to control group.

To examine the therapeutic potential of targeting the ATX/LPA axis in RA, BrP-LPA was injected intraperitoneally twice a week (following immunization boost; Fig. 4A), in groups of mice undergoing the development of collagen induced arthritis (CIA). The simultaneous pharmacological inhibition of ATX and antagonism of LPA receptors by BrP-LPA resulted in the development of arthritis in significantly fewer mice (Fig. 4B), as well as to reduced severity per arthritic limb (Fig. 4C). Histopathological analysis of the joints indicated marked decreased inflammation and synovial hyperplasia (Fig. 4D, E).

Importantly, this dual activity pharmacological agent showed no appreciable alternation of gross physiology (data not shown), indicating the absence of systemic side effects. Moreover, BrP-LPA administration did not interfere with the levels of anti-collagen antibodies (Fig. 4F). It has been previously reported that no significant differences in LPA levels in arthritic, ATX-overexpressing joints were detected [21], most likely due to the rapid LPA turnover [32] and the possible local delivery to its receptors by cell bound ATX [64]. Likewise, no changes in LPA (or LPC) levels were detected, in plasma or joint tissue of treated mice at the end of the monitoring period (three days after the last injection; data not shown).

Similar therapeutic results were also obtained when the BrP-LPA injection dosing regimen was increased to three times per week as well as to daily dosing (data not shown); however, the lower, biweekly dosage scheme was generally employed as it has been previously reported to be effective *in vivo* in an orthotopic breast cancer xenograft model [34]. Since biweekly injections of BrP-LPA were able to prevent the arthritic symptoms despite its PK profile, it appears that pulsatile inhibition of the local ATX/LPA axis is sufficient for disease management. Moreover, the metabolically stabilized α-halo phosphonate nature of BrP-LPA suggests that BrP-LPA may have longer drug-residency times. Indeed, BrP-LPA levels were found to persist for longer in joint tissue, as opposed to liver tissue and plasma (data not shown).

It was recently reported that pharmacological antagonism of LPA receptors 1 and 3 with ki16425 [78], previously shown to inhibit LPA-mediated COX-2 expression from SFs [70], resulted in a reduction of arthritis severity in the K/BxN serum transfer RA model [79] despite its very short half-life [80], suggesting that antagonism of LPA receptors, one of the properties of BrP-LPA, can be therapeutic. On the other hand, pharmacological potent ATX inhibition with GWJ-A-23 [81] during CIA development, although exhibiting clear therapeutic effects, was associated with severe side effects resulting in increased lethality (data not shown). GWJ-A-23 is an a-substituted phosphonate analog of S32826 [82] with increased solubility recently shown to attenuate ATX-driven pulmonary inflammation and fibrosis without exhibiting any side effects [83]. However, treatment with GWJ-A-23 during CIA was twice as long, urging caution (at least in the context of this animal model) on the potential side effects of long-term, potent ATX inhibition. On-going genetic studies of inducible ATX ablation in adult mice is expected to identify any pathological symptoms associated with potent (>>50%) ATX inhibition and investigate the appropriateness of ATX as a therapeutic target.

The exact mode of action of BrP-LPA, moderate ATX inhibition and/or LPA receptor antagonism or both (or other undetermined off site effects), in the context of disease pathogenesis remains to be elucidated. Nonetheless, the future commercialization of novel, potent specific inhibitors for ATX and antagonists for LPA receptors [33], [84] would undoubtedly aid in fine tuning the well-established therapeutic potential of targeting the ATX/LPA axis in RA and other inflammatory disorders.

## Supporting Information

Figure S1 **Analysis of ATX kinetics and** ***in vitro*** **characterization of BrP-LPA inhibition with different recombinant ATX proteins and different ATX activity assays.** **A–B.** The hydrolysis of LPC (16:0, 18:0) by R&D recombinant ATX protein measured with TOOS reagent and **C–D**. The hydrolysis of LPC (14:0, 18:0) measured with Amplex Red reagent and Echelon recombinant ATX follows Michaelis Menten kinetics. **E.** [FS-3] dependence of the steady state FS-3 hydrolysis rate by Echelon recombinant ATX, measured with FS-3 activity assay. The solid line represents the best fit to a rectangular hyperbola. **F–H**. Dose-response curves in the presence of various BrP-LPA concentrations (0.01–100 μM), show the percent residual ATX activity (% ATX activity) of Echelon recombinant protein, measured with (**F, G**) Amplex Red assay by choline release from different LPC species (50 μΜ); (F) 14:0 LPC; (**G**) 18:0 LPC and with H. FS-3 activity assay by the use of fluorescent lipid substrate FS-3. The presented values are the means (±std) of two independent experiments. The log concentration of BrP-LPA is used for the sigmoidal dose response curves.
(TIF)
