# Supplementary figures and images for: A Metabolically-Stabilized Phosphonate Analog of Lysophosphatidic Acid Attenuates Collagen-Induced Arthritis
Source: PLoS One. 2013 Jul 29;8(7):e70941. doi: 10.1371/journal.pone.0070941 (PMC3726599; doi:10.1371/journal.pone.0070941)

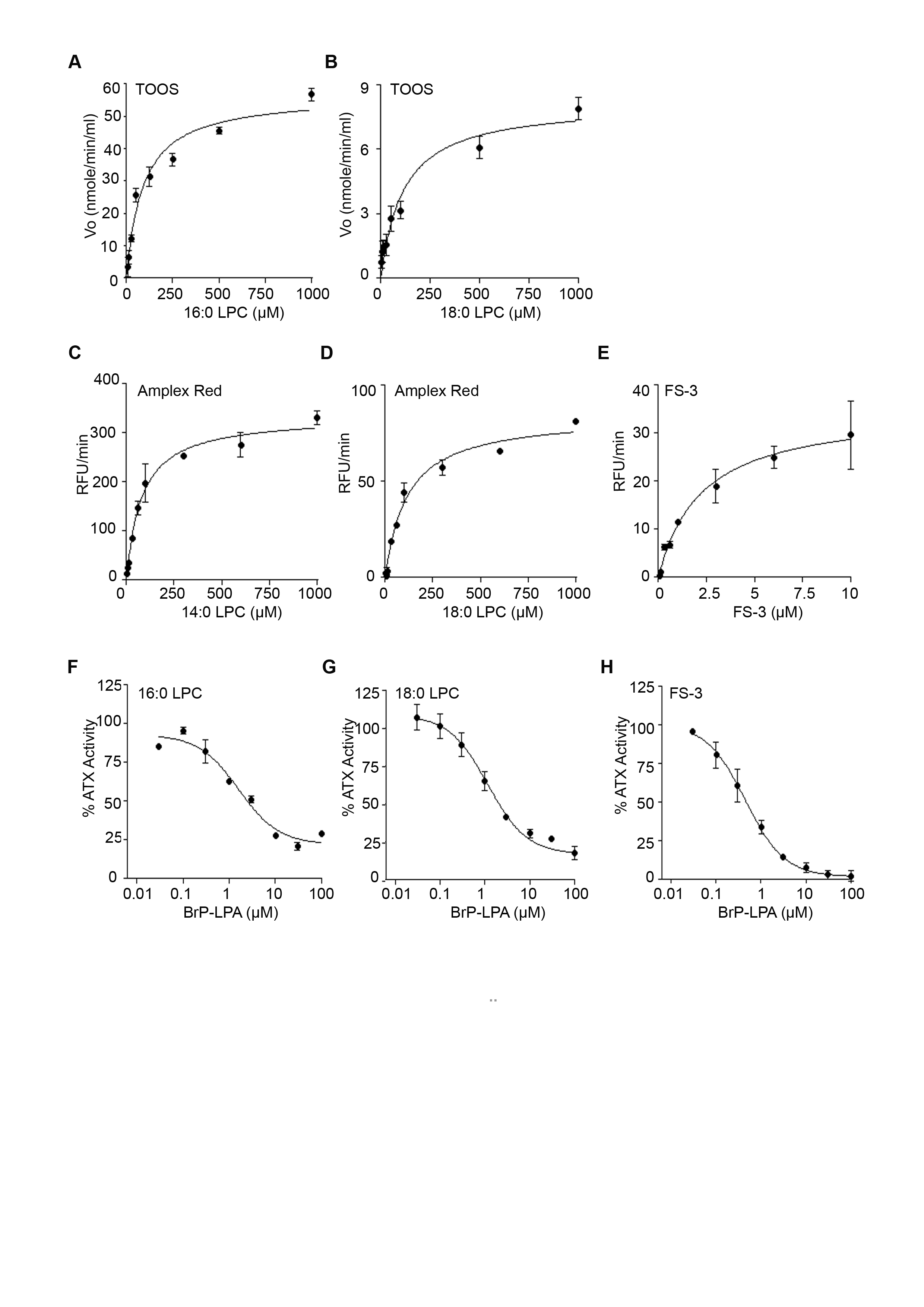

Supplement: Figure S1 — Analysis of ATX kinetics and in vitro characterization of BrP-LPA inhibition with different recombinant ATX proteins and different ATX activity assays. A–B. The hydrolysis of LPC (16:0, 18:0) by R&D recombinant ATX protein measured with TOOS reagent and C–D. The hydrolysis of LPC (14:0, 18:0) measured with Amplex Red reagent and Echelon recombinant ATX follows Michaelis Menten kinetics. E. [FS-3] dependence of the steady state FS-3 hydrolysis rate by Echelon recombinant ATX, measured with FS-3 activity assay. The solid line represents the best fit to a rectangular hyperbola. F–H. Dose-response curves in the presence of various BrP-LPA concentrations (0.01–100 μM), show the percent residual ATX activity (% ATX activity) of Echelon recombinant protein, measured with (F, G) Amplex Red assay by choline release from different LPC species (50 μΜ); (F) 14:0 LPC; (G) 18:0 LPC and with H. FS-3 activity assay by the use of fluorescent lipid substrate FS-3. The presented values are the means (±std) of two independent experiments. The log concentration of BrP-LPA is used for the sigmoidal dose response curves. (TIF) [file pone.0070941.s001.tif]
